# Comprehensive data analysis of human ureter proteome

**DOI:** 10.1016/j.dib.2016.01.050

**Published:** 2016-02-03

**Authors:** Sameh Magdeldin, Yoshitoshi Hirao, Amr El Guoshy, Bo Xu, Ying Zhang, Hidehiko Fujinaka, Keiko Yamamoto, John R. Yates, Tadashi Yamamoto

**Affiliations:** a Biofluid Biomarker Center (BB-C), Institute for Research Collaboration and Promotion, Niigata University, Niigata, Japan; b Department of Physiology, Faculty of Veterinary Medicine, Suez Canal University, Ismailia 41522, Egypt; c Department of Chemical Physiology, The Scripps Research Institute, 10550 North Torrey Pines Road, SR11, La Jolla, CA 92037, United States; d Biotechnology Department, Faculty of Agriculture, Al-Azhar University, Cairo 11682, Egypt

## Abstract

Comprehensive human ureter proteome dataset was generated from OFFGel fractionated ureter samples. Our result showed that among 2217 non-redundant ureter proteins, 751 protein candidates (33.8%) were detected in urine as urinary protein/polypeptide or exosomal protein. On the other hand, comparing ureter protein hits (48) that are not shown in corresponding databases to urinary bladder and prostate human protein atlas databases pinpointed 21 proteins that might be unique to ureter tissue. In conclusion, this finding offers future perspectives for possible identification of ureter disease-associated biomarkers such as ureter carcinoma. In addition, Cytoscape GO annotation was examined on the final ureter dataset to better understand proteins molecular function, biological processes, and cellular component. The ureter proteomic dataset published in this article will provide a valuable resource for researchers working in the field of urology and urine biomarker discovery.

## Specifications table

**Table:** 

Subject area	*Biology*
More specific subject area	*Proteomics – human proteome project*
Type of data	*Text files, raw mass spectrometry files*
How data was acquired	*Mass spectrometry*
Data format	*Raw, filtered, and, analyzed,*
Experimental factors	*Sample was prefractionated using OFFGel fractionator (Agilent technologies) prior to mass analysis*
Experimental features	*First released comprehensive date of human ureter proteome*
Data source location	*Niigata city, Japan*
Data accessibility	*Peptide Atlas link: ftp://PASS00641:ZI6249ae@ftp.peptideatlas.org/ PRIDE project #: PXD002620*

## Value of the data

• First human ureter proteome dataset.
• Integration to human proteome project data.
• Useful for urologist and better understanding of urogenital tract pathophysiology.

## 1 Data

Dataset presented in this article summarize the human ureter proteome. Whole tissue protein was extracted from healthy ureters and subjected to OFFGel prefractionation [1], [2], [3], [4], [5] to simplify sample complexity. The use of OFFGel prefractionation enabled deep proteome mapping of confident proteins. Next, generated ureter proteome was compared qualitatively to other databases to generate protein candidates associated with ureter tissue.

## 2 Experimental design, materials and methods

**Fig. 1:**
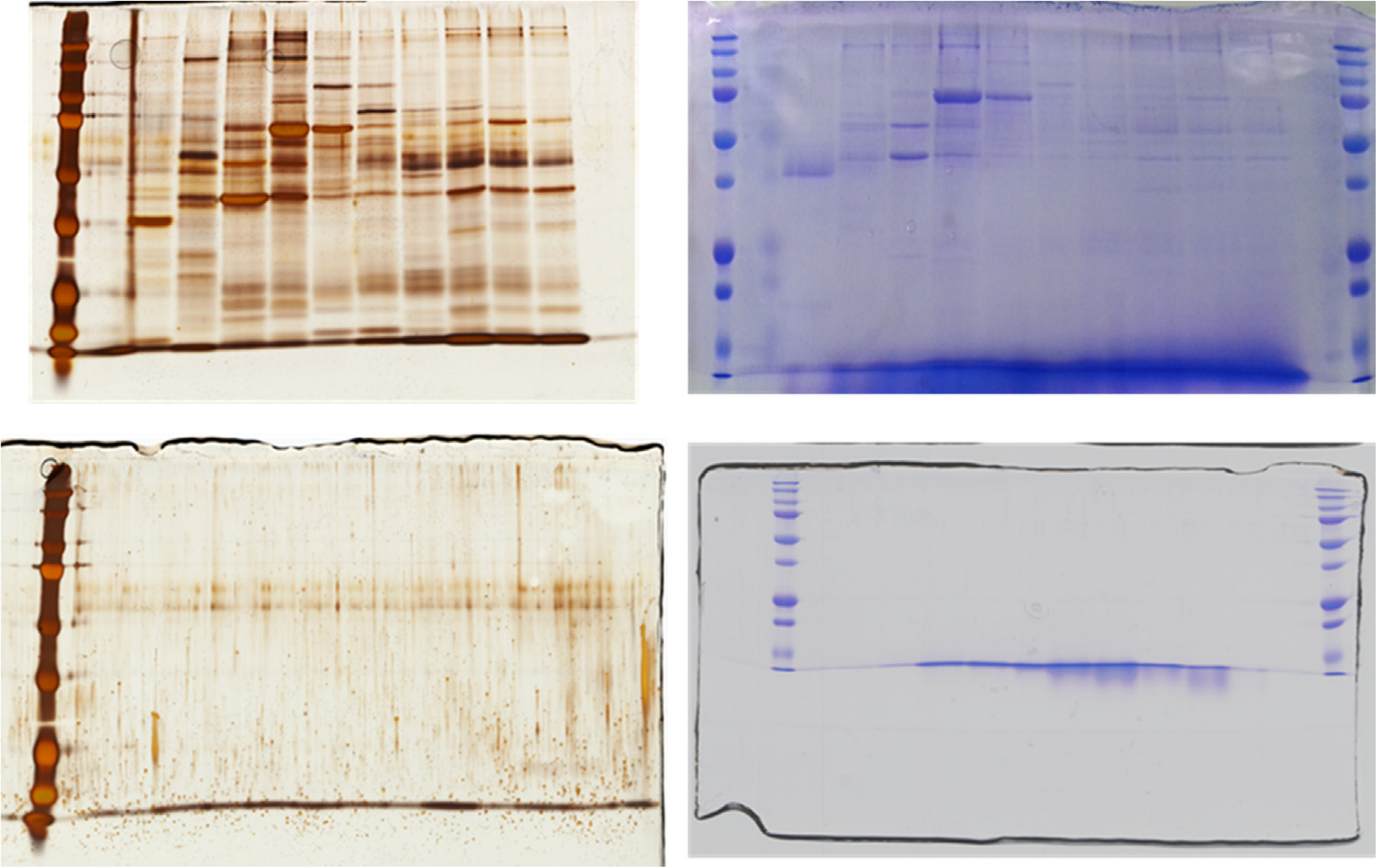
Clockwise from top left: silver stained SDS-PAGE of protein OffGel fractionation, stained with Commassie brilliant blue (R-250), silver stained SDS-PAGE of tryptic peptides, stained with Commassie brilliant blue (R-250).

Normal healthy ureter biopsy sample (4–5 cm length) was obtained from individuals with informed consent and under the approval of Committee of Ethics for Life and Genes of the Graduate School of Medical and Dental Sciences, Niigata University. Protein extracts were obtained by placing dissected ureter tissues in protein OFFGel prefractionation buffer supplied by the manufacturer (containing urea, thiourea, DTT, glycerol, and buffer with ampholytes pH (3–10)). Complete ultra-proteases (Roche, Mannheim) was added to the buffer. Precellys 24 tissue homogenizer was used for protein extraction at 4 °C. (Precellys, Bertin technologies). 2 mg of recovered protein extract was subjected to OFFGel prefractionation as previously described [6]. Following successful prefractionation, acetone precipitation, and protein quantification, 80 µg from each OFFGel fractions (*n*=12) were subjected to reduction and alkylation, and digested with trypsin as described elsewhere [7], [8]. Digested peptide solution was acidified using 90% formic acid to a final pH 3 and enriched using stage tip15, 16. Efficiency of fractionation and digestion was confirmed as shown in Fig. 1. Chromatography of purified peptides was performed using Thermo Q-Exactive and separation was applied using a binary gradient for 120 min with acetonitrile as mobile phase. The precursor full MS scan ranged from 40 to 1200 *m*/*z*. Dynamic exclusion setting used were as follows: repeat count, 1; repeat duration, 30 s; exclusion list size, 450; and exclusion duration 60 s. All raw data (Thermo.RAW) are available in Peptide Atlas repository at ftp://PASS00641:ZI6249ae@ftp.peptideatlas.org/.

**Fig. 2:**
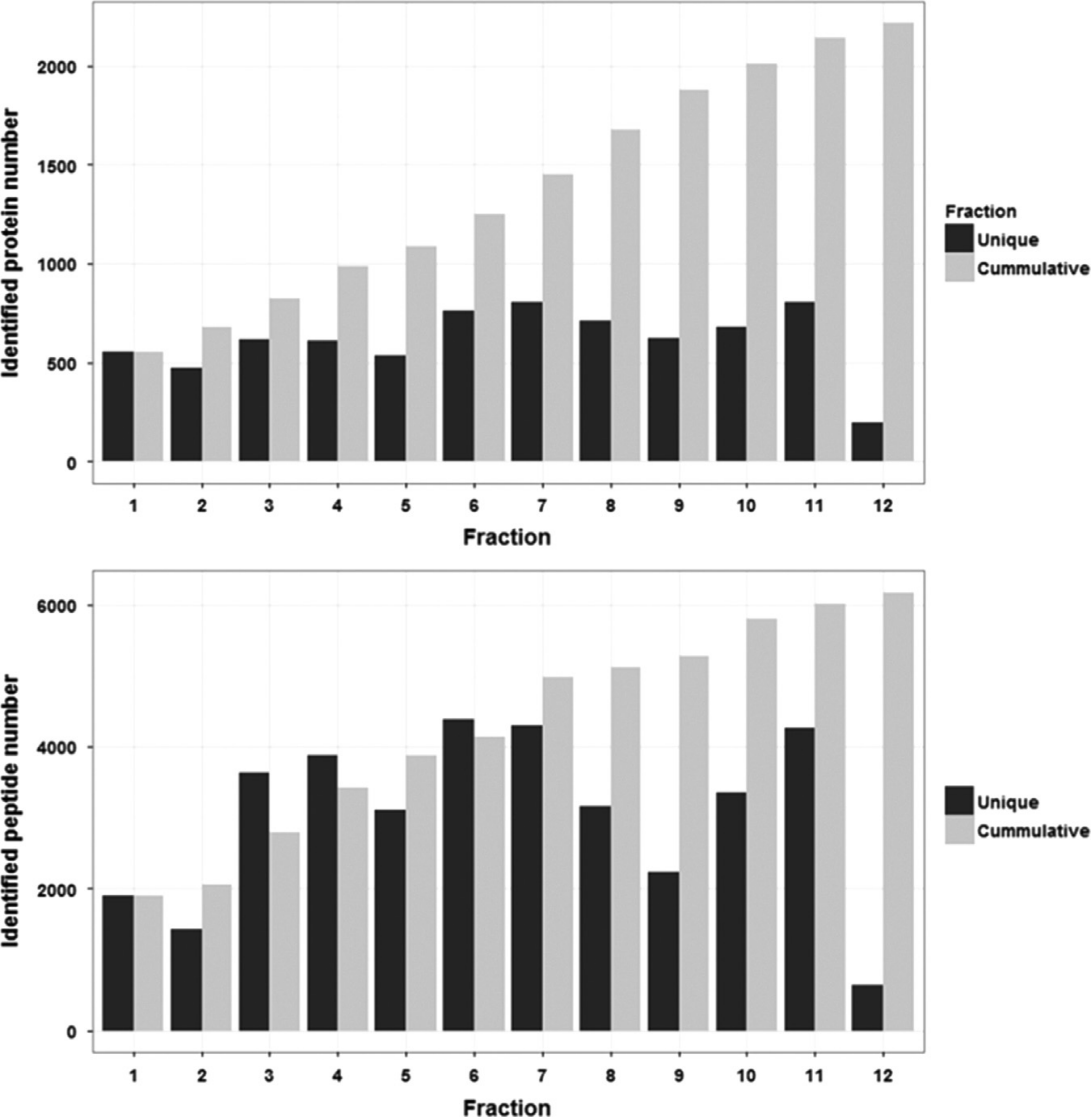
Identified proteins and peptides of the human ureter proteome. Upper figure, identified non-redundant proteins (within fraction) in 12 OffGel fractions. Lower, identified non-redundant peptides (within fraction) in 12 OffGel fractions. Black bars represent unique protein or peptide candidate within fraction. Gray bars represent newly added proteins/peptides from the subsequent fraction.

**Fig. 3:**
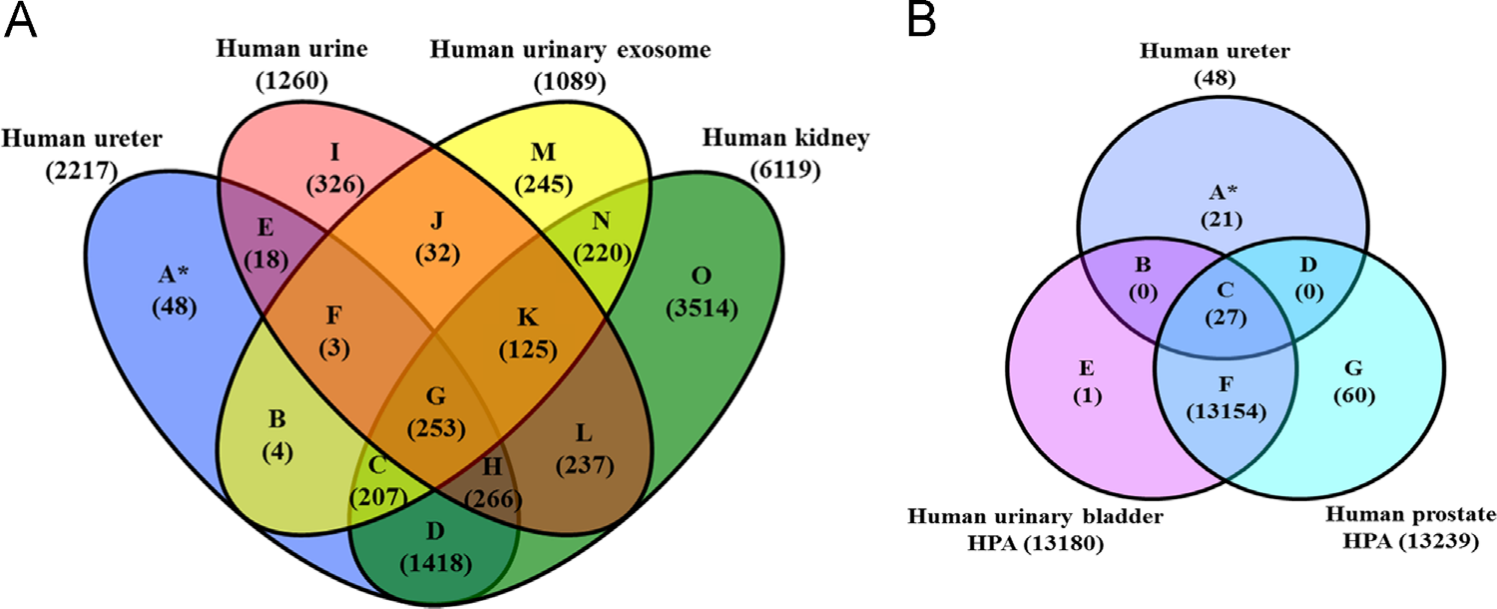
Venn diagrams of unique and shared ureter proteome with other databases. (A) Venn diagram of human ureter proteome overlapped with urinary, urinary exosomal, and kidney mass spectrometric databases. (B) Non-shared proteins in panel A (zone A) was further compared to human urinary bladder and prostate databases retrieved from human protein atlas (based on immunohistochemistry) where 27 proteins were shared and 21.

**Fig. 4:**
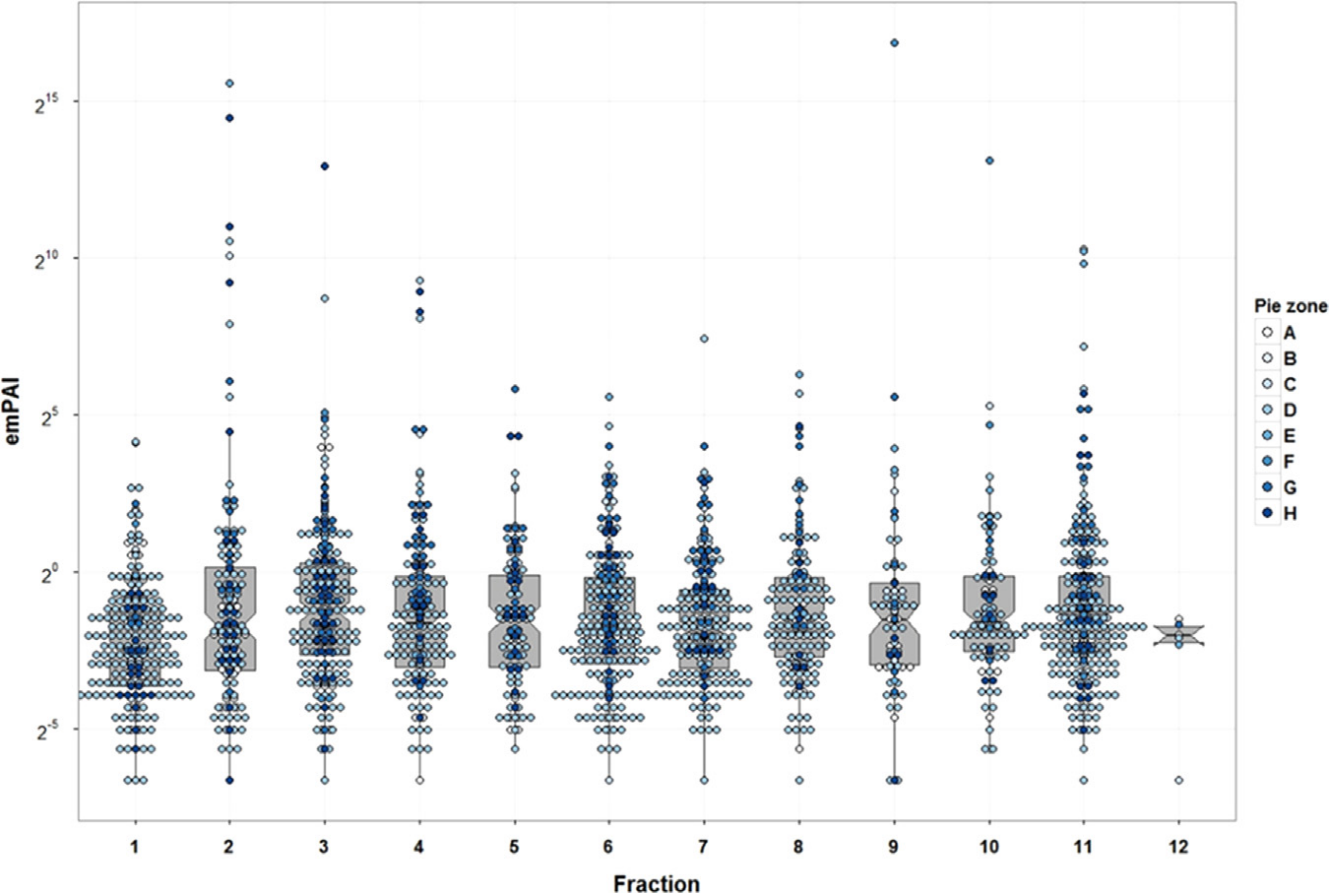
Whisker and box plot overlaid with dot plot showing median and quartile values of the Modified Protein Abundance (emPAI) for ureter proteome database. Color dots represent pie zone location based on Fig. 2A sorting. *Y* axis shows emPAI value in log2 view.

Protein and peptide identification were searched by MASCOT in addition to ProluCID search engine implemented in the integrated proteomics pipeline; IP2 (http://integratedproteomics.com/, version 1.01). Tandem mass spectra were generated using RawExtract (Ver 1.9.9) and the MS/MS spectra were searched against updated UniProtKB/TrEMBL (*Homo sapiens*, 935,651 entries). The spectral search space included all fully and half tryptic peptide candidates within a 50-ppm window surrounding the peptide candidate precursor mass. Carbamidomethylation (+57.02146) of cysteine was considered a static modification and oxidation at M, HW (+15.995) as variable modification. Peptide candidates were filtered to 0.1% FDR and proteins candidates to 1% FDR using DTASelect with a 10-ppm (Fig. 2, Fig. 3, Fig. 4

## Conflicts of interest

The authors have declared no conflict of interest.

## Funding

This work was supported by the Center of Innovation Program from Japan Science and Technology Agency, JST to TY and a Grant-in-Aid for scientific research (B) from Japan Society for Promotion of Science, JSPS to SM (23790933), and standard JSPS grant for foreign researcher (P 14105) from Ministry of Education, Culture, Sports, Science and Technology of Japan to SM. The funders had no role in study design, data collection and analysis, decision to publish, or preparation of the manuscript.
