# Supplementary material for: Comprehensive data analysis of human ureter proteome
Source: Data Brief. 2016 Feb 3;6:853–7. doi: 10.1016/j.dib.2016.01.050 (PMC4749945; doi:10.1016/j.dib.2016.01.050)

**Conflict of interest**

We confirm that the manuscript has been read and approved by all named authors and that there are no other persons who satisfied the criteria for authorship but are not listed. We further confirm that the order of authors listed in the manuscript has been approved by all of us.

We confirm that we have given due consideration to the protection of intellectual property associated with this work and that there are no impediments to publication, including the timing of publication, with respect to intellectual property. In so doing we confirm that we have followed the regulations of our institutions concerning intellectual property.

Sameh Magdeldin (on behalf of all co-authors in the article)

1/21/2016


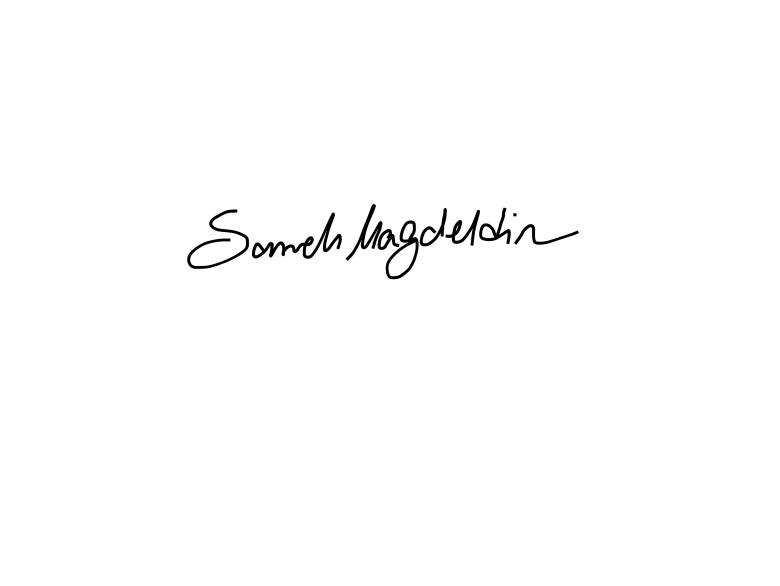

Supplement: Supplementary file 1 — Supplementary material [file mmc1.docx]
